# SSR Mapping of QTLs Conferring Cold Tolerance in an Interspecific Cross of Tomato

**DOI:** 10.1155/2016/3219276

**Published:** 2016-07-19

**Authors:** Yang Liu, Tengxia Zhou, Haiyan Ge, Wen Pang, Lijie Gao, Li Ren, Huoying Chen

**Affiliations:** School of Agriculture and Biology, Shanghai Jiao Tong University, 800 Dongchuan Road, Minhang District, Shanghai 200240, China

## Abstract

A population of 146 RILs (Recombinant Inbred Line) was derived from the cross between a cold-sensitive cultivated* Solanum lycopersicum* L. XF98-7 and a cold-tolerant wild* Solanum pimpinellifolium* LA2184. Relative germination ratio (RGR) and chilling index (CI) were used to evaluate the cold tolerance of the parental lines and RILs. It was found that the RGR and CI were significantly different between* S. lycopersicum* XF98-7 and* S. pimpinellifolium* LA2184 under cold treatment, indicating that wild species was more adapted to chilling temperature. The continuous and normal distribution of RGR and CI in RIL population suggested that the trait of cold tolerance was a typically quantitative trait controlled by multigenes. The molecular linkage map was constructed by using 120 simple-sequence repeat (SSR) markers, resulting in 15 linkage groups, with a total distance of 256.8 cM and average interval of 2.14 cM. Five QTLs controlling RGR and four QTLs for CI were detected with genetic contribution ranging from 0.95% to 19.55%. Thus, the nine QTLs will provide references for further fine position mapping for cold tolerance. The polymorphic markers could be used as a way of indirectly selecting the plant trait of interest and would promote developing new tomato variety by marker-assisted selection.

## 1. Introduction

Tomato (*Solanum lycopersicum* L.), one of the most widely cultivated vegetable crops, was originated from South America. Its rich nutrients and unique flavor promote health benefits to human. As a model plant, tomato also plays an important role in modern molecular biology science and genetic breeding research.

Tomato is a thermophilic vegetable and sensitive to low temperature. With the optimal growth temperature of 25°C, the germination and vegetative growth are inhibited when temperature is lower than 10°C and irreparable damage would occur under 6°C [1]. Thus, low temperature becomes one of the main restrictions for the yield improvement and geographic expansion in tomato. Breeding experts have been aiming at developing cultivated tomato with improved cold tolerance [2]. Thanks to the wild species that survive in suboptimal environment, they become the natural germplasm repository with abundant genetic diversity, allowing us to select the desirable or required characteristics, such as resistance to cold, drought, and diseases in tomato [3]. For example, wild species of* S. habrochaites* and* S. pimpinellifolium* growing in high-attitude regions often encounter chilling temperature at night, but they can survive and thrive during their growing seasons [4, 5]. Walker et al. found cold tolerance segregation in the BC_1_F_2_ population from the cross of cultivated tomato and* S. habrochaites* by investigating the chlorophyll fluorescence [6]. Foolad et al. also discovered the segregation in the BC_1_S_1_ population derived from cultivated tomato and* S. pimpinellifolium* by investigating seed germination under low temperature [7, 8].

The characteristic of cold tolerance in tomato is a quantitative trait, correlated with multigenes. Some quantitative trait loci (QTLs) for cold tolerance have been identified by using polymorphic molecular markers. The cold tolerance of a BC_1_ population from the parental lines of* S. lycopersicum* NC84173 and* S. pimpinellifolium* LA722 was assessed, and three QTLs on chromosome 1 and two on chromosome 4 were detected by using 151 RFLP markers [7]. Using a BC_1_ population between cultivated tomato T5 and wild accession LA1778, six QTLs were found to be linked to the shoot wilting after 2 hours of exposure to chilling temperature (4°C) of the population's root [9]. The QTL mapping from BC_3_S_1_ population derived from* S. lycopersicum* 9706 and* S. pimpinellifolium* LA722 suggested that five QTLs were linked to seed germination and two related to vegetative growth for cold tolerance, with genetic contribution from 3.3% to 32.9% [10]. The root chilling experiment with a RILs population derived from cultivated tomato T5 and wild tomato LA1778 mapped a major QTL on chromosome 9 responsible for shoot turgor maintenance [11].

In this study, a RIL population, generated from the cross of* S. lycopersicum* XF98-7 and* S. pimpinellifolium* LA2184, was used to locate quantitative trait loci for cold tolerance by investigating the relative germination rate (RGR) and chilling index (CI) under low temperature conditions.

## 2. Materials and Methods

### 2.1. Plant Material

146 RIL individuals (F_8_) were developed from the cross* S. lycopersicum* XF98-7 (cultivated and cold-sensitive tomato) and* S. pimpinellifolium* LA2184 (wild and cold-tolerant tomato).* S. lycopersicum* XF98-7 and* S. pimpinellifolium* LA2184 were obtained from Shanghai Jiao Tong University in China and University of California in USA. The RIL population was constructed by Tomato Research Team from School of Agriculture and Biology of Shanghai Jiao Tong University.

When 4-5 true leaves appeared, leaf tissue from the RIL population and parental lines were collected for total genomic DNA extraction, which was used to identify molecular markers linked to the trait of interest.

### 2.2. The Evaluation of Cold Tolerance for Parental Lines and RIL Population

100 random seeds of the parental lines and the RIL population were selected, sown on sterile petri plate that had wet filter papers on the bottom, and then placed into the dark incubators. The control samples were maintained under 25 ± 1°C, while the treated samples were kept at 11 ± 1°C. We used three biological replicates for RIL population and four for the parental lines. The amount of seed germination was recorded for 28 consecutive days after sample treatment, and the total seed germinating in the 28 days was available to calculate the germination ratio. The germination ratio (GR) and relative germination ratio (RGR) were used to estimate the cold tolerance of the individuals. A high RGR indicated a strong low temperature tolerance, which was calculated by the equation of RGR = (RG  in  cold  treatment/RG  in  control  treatment) × 100%.

After germination, 12 seedlings of the parental lines and RIL population were planted in 72-cell flats. At the stage of 4-5 true leaves, the plants were transferred to incubators with the light cycle of 12 h days/12 h nights and chilling temperature at 2 ± 1°C. After 48 hours, the chilling injuries of each individual by using chilling indexes (CI) were estimated for three replicates of RILs and four for parental lines. The injuries of the plants were visually scored based on the leaves and shoots wilting with 0 to 4 scale criterion. Score of 0 meant there were normal leaves and shoots on plants. Score of 1 meant the plant had only few flaccid leaf tips. Score of 2 represented that approximately 50% leaf tips were flaccid, but the main shoots were in normal condition. Score of 3 represented that more than 50% leaf tips were flaccid and shoots were wilted. Score of 4 described that the plant completely died. We used CI value to measure the cold tolerance by the equation of CI = ∑*Xa*/(*n*∑*X*) = (*X*_1_ *a*_1_ + *X*_2_ *a*_2_ + ⋯+*X*_*n*_ *a*_*n*_)/*nT*.*X*_*n*_ was the number of the plants of each injury score, and *a*_*n*_ stands for the score of the injury. *T* was the total number of investigated plants. A low CI indicated that the leaves and shoots were less injured and strongly tolerant for chilling temperature.

### 2.3. Date Analysis Procedures

The SPSS Statistics software version 20.0 (IBM Corp., Armonk, NY, USA) was used to analyze data variance, normal distribution, and correlation coefficient between variables.

### 2.4. DNA Extraction and SSR Reaction

The CTAB method of DNA extraction for 146 RILs and parental lines was used following the protocol of Murray and Thompson [12].

The sequence of 1183 primer pairs for SSR markers was obtained from the Sol Genomics Network (https://solgenomics.net/). The primers were synthesized by GenScript (Nanjing) Co., Ltd. The polymerase chain reaction (PCR) with a total volume of 20 *μ*L contained 50 ng DNA template, 2.0 mM Mg^2+^, 1x PCR buffer, 0.2 mM dNTPs mixture, 100 nM forward and reverse primers, and 1 U Taq DNA polymerase. The thermal program for PCR was an initial denaturation for 3 min at 94°C, denaturation for 30 s at 94°C, annealing for 45 s at 55–60°C, extension for 60 s at 72°C for 35 cycles, and an extra extension for 10 min at 72°C.

The PCR amplification products were separated by 5–8% vertical polyacrylamide gels and silver stained following the method of Zhang et al. [13].

### 2.5. Construction of Linkage Map and QTL Analysis

In this study, the molecular linkage map was constructed by JionMap 4.0 software with a LOD value 3.0 as the threshold. The map genetic distance (cM) was calculated in Kosambi function.

The location and effects of the QTLs were detected in composite interval mapping (CIM) method by using Windows QTL Cartographer version 2.5 software, and a minimum LOD score of 2.0 was set to indicate the presence of QTLs. The QTL nomenclature followed McCouch et al. [14].

## 3. Result

### 3.1. Chilling Tolerance of Parental Lines and RIL Population

The parental lines showed significant difference in RGR and CI in this study (Table 1). The RGR of* S. pimpinellifolium* LA2184 was significantly higher than that of* S. lycopersicum* XF98-7, and the CI of* S. pimpinellifolium* LA2184 was significantly lower than* S. lycopersicum* XF98-7's. The data indicated that* S. pimpinellifolium* LA2184 was much more cold tolerant than* S. lycopersicum* XF98-7, which was consistent with the previous assumption that wild species was more adapted to chilling temperature.

**Figure 1:**
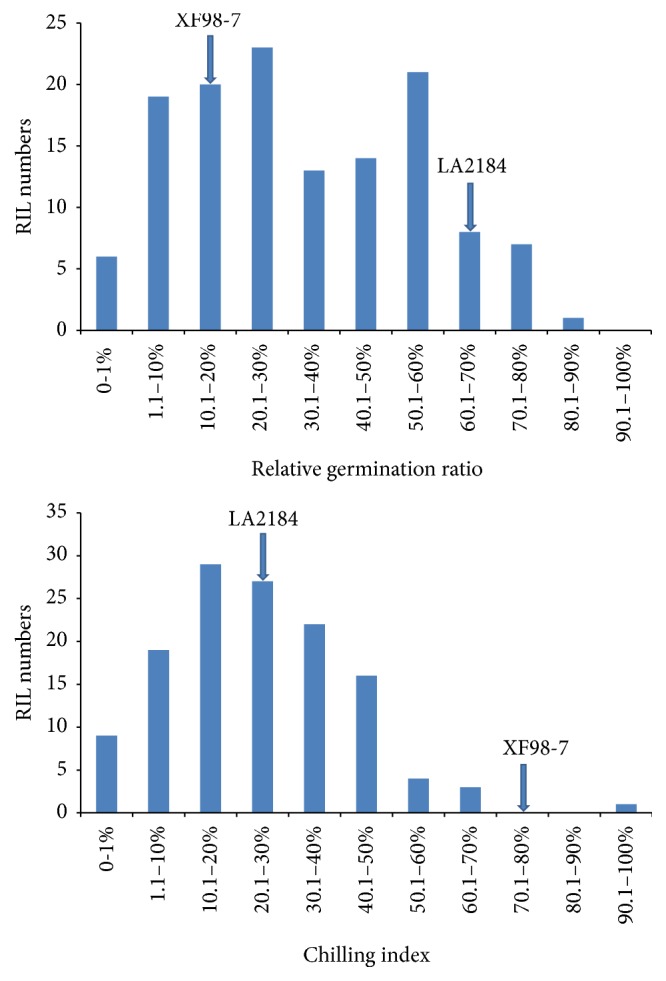
Frequency distribution of relative germination ratio and chilling index associated with cold tolerance in parental lines and RIL population.

Continuous distribution was observed in RGR and CI trait among the RIL population (Figure 1). The Skewness and Kurtosis absolute values (0.327 and 0.902) of RGR trait in RIL were less than 1.0, so RGR followed approximately normal distribution, and so was the CI trait (with Skewness = 0.382, and Kurtosis = 0.487). The data of the phenotypes also showed an obvious transgressive segregation, 23.3% of the RIL population expressed lower RGR than* S. lycopersicum* XF98-7, and 7.5% of RIL exhibited higher RGR than* S. pimpinellifolium* LA2184, and 44.52% demonstrated lower CI than* S. pimpinellifolium* LA2184.

The correlation analysis between RGR and CI showed that there was no significant correlation at a given significance level *P* = 0.05. The result indicated the cold tolerance of tomato during seed germination and vegetative growth was independent probably due to the different genetic background.

### 3.2. Genotype Analysis and Linkage Map Construction

**Figure 2:**
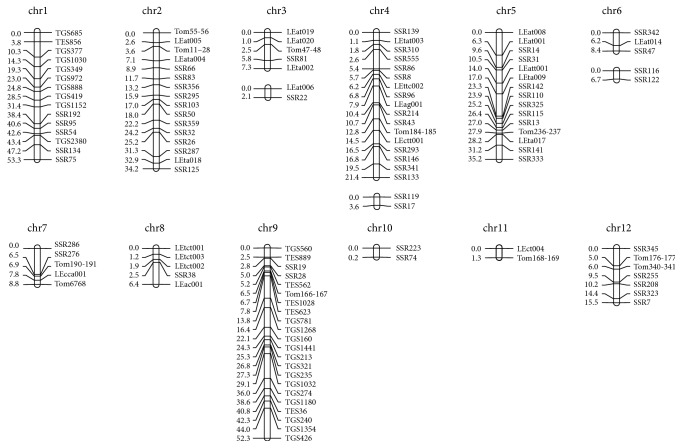
The genetic linkage map for SSR makers in RIL population.

A total of 1183 SSR primers were screened in parental lines and 142 primers (12.0%) were polymorphic. The informative SSR markers were continued to genotype RIL population. A molecular linkage map was built based on 120 SSR markers with a threshold value of 3.0 by using JoinMap 4.0 software (Figure 2). The molecular linkage map contained 15 linkage groups, with two groups on chromosomes 3, 4, and 6 and one group on other chromosomes. The total distance of the linkage map was 256.8 cM with an average interval distance of 2.14 cM. The range of genetic distance between two markers was from 0.18 cM to 8.30 cM.

### 3.3. Identification of QTL Associated with Cold Tolerance

The QTLs analysis was conducted by Windows QTL Cartographer version 2.5 software by using CIM method. Nine QTLs were mapped, linked to the cold tolerance of tomato, and distributed on chromosomes 1, 2, 3, 4, 9, and 12. The genetic contribution of single QTL ranged from 0.95% to 19.55% (Table 2).

There were five QTLs related to RGR, with two located on chromosome 1 and the other three on chromosomes 4, 9, and 12. The biggest and smallest genetic contributions were 19.55% and 5.95%. Except qRGI-4-1 with an additive effect 0.0763, all the positive genes were contributed from* S. lycopersicum* XF98-7. Four QTLs related to CI were observed on chromosomes 1, 2, 3, and 9, responsible for 28.91% of the total phenotypic variation. The positive genes of qCI-1-1 and qCI-3-1 were donated from* S. pimpinellifolium* LA2184, and the genes of qCI-2-1 and qCI-9-1 were from* S. lycopersicum* XF98-7.

## 4. Discussion

In spite of different evaluation criteria, we used the value of RGR and CI on a RIL population derived from the cross of* S. lycopersicum* XF98-7 and* S. pimpinellifolium* LA2184. There was a significant separation for the seed germination rate and CI on RILs [10, 15, 16]. We constructed a molecular linkage map from 120 SSR markers and also detected QTLs related to cold tolerance in tomato. The result revealed that the RGR and CI were significantly different between the parental lines, with a higher RGR and a lower CI for the cold-tolerant wild species of* S. pimpinellifolium* LA2184 than the cold-sensitive cultivated species of* S. lycopersicum* XF98-7. The continuous and normal distribution observed in RGR and CI among RIL population indicated that the trait of cold tolerance was a typically quantitative trait controlled by multigenes. It was surprising that there were few RILs with CI trait similar to* S. lycopersicum* XF98-7. We checked the fruits of RILs and found a partial separation, too. The fruit phenotype tend to be like* S. pimpinellifolium* LA2184, and there were much more small fruits. We assumed it was because of the strong resistance and high ratio of fruit set of* S. pimpinellifolium* LA2184; more small fruit-plants were selected when the RIL population was built. There was no significant correlation between RGR and CI in RILs, which was consistent with the study done by Foolad [17].

Genetic markers have made the process of plant breeding more efficient and greatly promoted genetic research in plants, especially for QTL mapping to find genetic variation contributed to a trait we are interested in. Foolad et al. took advantage of polymorphic markers and found five QTLs associated with seed germination in low temperature on chromosomes 1 and 4 from the BC_1_S_1_ population derived from* S. lycopersicum* NC84173 and* S. pimpinellifolium* LA722 [7]. Three major QTLs on chromosomes 4, 8, and 9 and six minor QTLs on chromosomes 1, 7, 9, 11, and 12 were found to be linked to seed rapid germination in low temperature [18]. Another five QTLs from chromosomes 1, 2, 5, and 11 were also detected to be associated with cold tolerance [10]. In this study, five QTLs involved in RGR were identified on chromosomes 1, 4, 9, and 11. In summary, QTL conferring cold tolerance on chromosome 1 was overlapped from the 4 studies above and our detection that QTLs on chromosomes 4, 9, and 11 were consistent with that in Foolad's studies [17]. The possibility of QTLs linked to cold tolerance located on chromosomes 1, 4, 9, and 11 provides references for fining position mapping and the genetic markers would promote developing new crop variety by marker-assisted selection.

Four QTLs conferring CI were detected on chromosomes 1, 2, 3, and 9, with the contribution of single QTL to phenotypic variation between 0.95% and 10.34%. There was neither correlation between the RGR and CI phenotype data in RIL population nor colocalization found for the two traits, which indicated that cold tolerance of tomato during seed germination and vegetative growth is controlled by different genes [19]. Vallejos and Tanksley found QTLs on chromosomes 6, 7, and 12 that affected the cold tolerance of tomato plants [20]. Liu et al. located two QTLs related to cold resistance in young plants on chromosomes 2 and 8, in which the genetic contributions were 3.3% and 4.9% [10]. Goodstal et al. identified a major QTL for shoot turgor maintenance when root was exposed to chilling temperature. Despite multiple QTLs conferring cold tolerance in vegetative growth, their locations were independent almost across the whole genome which lead to low genetic contribution to the variant phenotype [21]. And we can see that the results all above differ from each other; no QTLs locate in the same region. Except for the material difference in the studies, the density of the molecular linkage map also influenced the number of and the related QTLs. A single QTL with high effect value would be broken into several minor QTLs when the marker density of the linkage map increases and the population size gets larger [11].

The past studies showed that the cold tolerance of tomato was a complex quantitative trait controlled by multiple genes, and genes that control the low temperature resistant trait during different developmental stages were not the same. Though much effort had been made to explain the genetic background of tomato response to cold stress and some related QTLs were mapped, the chromosome and the position where the QTLs located were quite different and the repeatability of the results was low. We assume that no uniform phenotypic assessment criteria are a main obstruction for QTLs detection besides the inherent complexity of cold resistance trait. For example, to evaluate the cold tolerance during seed germination, the time when germination rate reaches 25% (T25), 50% (T50), and 75% (T75) was induced by Foolad et al. [7, 22], while Zhao et al. [23] set the inhibition growth of radicles that recovered from cold stress for 3 days as a criterion. Liu et al. [10] adopted germination index (GI) and relative germination index (RGI) to assess tomato cold tolerance. More different standards to evaluate the cold tolerance were used, such as chlorophyll fluorescence, the degree of shoot wilting, root ammonium uptake, the ratio of shoot fresh weight, and the leaf and shoot injuries [6, 9, 16, 24]. Thus, the detection results of QTLs conferring to cold tolerance in vegetative growth were greatly variable and low repeatable. To have a profound understanding of cold tolerance in tomato and accelerate the process of developing commercial cultivars tomato with improved cold tolerance, a uniform standard to evaluate the chilling injuries should be established immediately.

## Figures and Tables

**Table 1:** Phenotype analysis of parental lines.

Materials	Relative germination ratio (E ± SD)	Chilling index (E ± SD)
XF98-7	0.137 ± 0.025^a^	0.713 ± 0.052^a^
LA2184	0.626 ± 0.088^b^	0.345 ± 0.050^b^

Note: different small letters in each column mean significant difference at 0.05 level.

**Table 2:** QTLs analysis for cold tolerance in RIL population using CIM method.

Trait	QTL	Chromosome	Position (cM)	Marker interval	LOD score	*R* ^2^ (%)	Additive effect
RGR	qRGI-1-1	1	40.4	SSR192 - SSR95^*∗*^	5.45	19.55	−0.1379
	qRGI-1-2	1	47.2	TGS2380 - SSR134^*∗*^	2.53	8.52	−0.0888
	qRGI-4-1	4	10.4	LEag001 - SSR214^*∗*^	2.06	6.02	0.0763
	qRGI-9-1	9	7.8	TES623^*∗*^ - TGS781	2.12	5.95	−0.0617
	qRGI-12-1	12	8.0	Tom340-341^*∗*^ - SSR255	4.26	11.33	−0.1143

CI	qCI-1-1	1	9.8	TES856^*∗*^ - TGS377	3.25	0.95	0.0208
	qCI-2-1	2	18.0	SSR50^*∗*^ - SSR359	2.96	10.34	−0.0828
	qCI-3-1	3	0.0	LEat019^*∗*^ - LEat020	3.01	10.31	0.0776
	qCI-9-1	9	26.8	TGS321^*∗*^ - TGS235	2.35	7.31	−0.0652

^*∗*^The closer molecular marker to the QTL position.
